# Worms

**Published:** 1872-08

**Authors:** 


### WORMS.

About thirty kinds in all have been found in the human body. As there are three descriptions of living things which infest the skin of man, — the head louse, the crab louse, and the body louse, so there are three kinds of living things which find their dwelling and feeding places in the interior, in the alimentary canal, — the pin-worm, the fish-worm, and the tape-worm.

### THE FISH-WORM, so called from its resemblance in shape, size, and color to the worm used for baiting hooks to catch fish, and which appear in such numbers when the ground is turned up in the garden for spring plantings, called technically *Ascaris lumbricoides*, or round worm, is mostly found in the small intestines, but sometimes finds its way into the large intestines and into the stomach, even coming up into the throat and out of the mouth ; and frequently have been from half a foot to half a yard long, and as large round as a gold pen. It is only when a good many are together that any special harm may be done. These worms get into the body by drinking water from shallow wells or muddy streams. Sometimes in adults they cause nausea, vomiting, and various symptoms of indigestion for weeks together, until they are vomited or voided. There is no symptom or set of symptoms which certainly indicates the presence of worms in adults or children, except the sight of the worm itself after it leaves the body, although in children they may be suspected when they are observed to pick the nose, grind the teeth in sleep, complain of itching at the lower outlet of the bowels, swollen belly, irregular appetite, and sometimes convulsions; but one or all of these symptoms may arise from several other ailments. So there is no really certain proof that an infant, child, or adult "has worms," except the sight of one expelled; then a physician should be called.

### PIN WORMS

are sometimes a source of intolerable discomfort, by causing an inappeasable itching at the exit of the rectum, especially when getting a little warm after first going to bed. They are called pin-worms by the common people, because they are almost as slender as a pin, and short, from a twelfth to half an inch long; sometimes they come away in incredible quantities; besides the itching, they sometimes cause a great deal of nervous irritation, and keep the whole system in a debilitated condition. Inject a tea-spoonful or two of camphor water, made by putting a piece of camphor into a small bottle; and pour on a teacupful of water; shake it and use it; the water may be added from time to time, until the camphor has disappeared. Or take a tea-spoonful of the tincture of *Cocculus Indicus* to a teacup half full of common water, and use it as an injection alone, or with the camphor water, making either stronger. Sometimes an injection of lime water, as obtained from a druggist, answers the purpose; or press mercurial ointment into the part, and retain it over night; or take twelve grains of santonine, and thin it with cocoa butter, enough to make four rolls an inch long, cone-shaped, and near as large as the little finger; introduce one of these into the rectum on going to bed every night. This is a valuable remedy, and seldom fails.

### TAPE-WORMS

have no sex originally, but if one of them gets into the human body in such a way that it can get some air, as in the alimentary canal, then it takes on sex, and reproduces itself. It has a head, with a short neck, to which is appended a kind of flat link; then comes another link, another and another, growing in length all the time, until there is a chain of three or four hundred links, or more. The links at the end of the tail are cast off, sometimes six or eight a day; the whole animal is twenty or thirty feet long. There are no symptoms certainly indicating their presence, except seeing one or more of these links in the dejections. When one or more of these segments have been observed, it will be found that certain symptoms have been more or less present, such as irregular appetite, disturbed sleep, despondency, sadness, dyspepsia, itching of the nose, and general nervous irritation. Not long since an apparatus was devised for fishing it out through the throat. Eating largely of

PUMPKIN SEEDS

has the credit of having cured several serious cases of tape-worm. Gourd seeds, peeled and made into a paste with honey, about two hundred at a time, taken fasting, as the pumpkin seeds are, have been efficient in a number of cases.

These remedies are more efficient if nothing has been eaten for twenty or twenty-four hours; this causes the worm to be ravenously hungry, and thus to take into the stomach what its instincts would otherwise forbid. Or make a strong tea of pumpkin or gourd seed, drink it at bed-time, plentifully, not having eaten anything whatever since breakfast; the next morning a strong cathartic will bring away the entire worm, dead or alive. In one case this brought a worm away, twenty-five feet long.

